# Polymorphisms in *CRYBB2* encoding βB2-crystallin are associated with antisaccade performance and memory function

**DOI:** 10.1038/s41398-020-0791-0

**Published:** 2020-04-21

**Authors:** Ina Giegling, Annette M. Hartmann, Just Genius, Bettina Konte, Stephan Maul, Andreas Straube, Thomas Eggert, Christoph Mulert, Gregor Leicht, Susanne Karch, Ulrich Hegerl, Oliver Pogarell, Sabine M. Hölter, Hans-Jürgen Möller, Jochen Graw, Dan Rujescu

**Affiliations:** 1 Department of Psychiatry, Psychotherapy and Psychosomatics, Martin-Luther-University Halle-Wittenberg, Halle, Germany; 2 UCB Biopharma S.P.R.L., Brussels, Belgium; 3 Department of Neurology, Ludwig-Maximilians-University, Munich, Germany; 4 Centre of Psychiatry and Psychotherapy, Justus-Liebig-University, Gießen, Germany; 5 Department of Psychiatry and Psychotherapy, University Medical Center Hamburg-Eppendorf, Hamburg, Germany; 6 Department of Psychiatry and Psychotherapy, Ludwig-Maximilians-University, Munich, Germany; 7 Department of Psychiatry, Psychosomatics and Psychotherapy, Goethe-University, Frankfurt, Germany; 8 Helmholtz Center Munich - National Research Center for Environmental Health, Institute of Developmental Genetics, Neuherberg, Germany

**Keywords:** Clinical genetics, Clinical genetics

## Abstract

βB2-crystallin (gene symbol: *Crybb2*/*CRYBB2*) was first described as a structural protein of the ocular lens before it was detected in various brain regions of the mouse, including the hippocampus and the cerebral cortex. Mutations in the mouse *Crybb2* gene lead to alterations of sensorimotor gating measured as prepulse inhibition (PPI) and reduced hippocampal size, combined with an altered number of parvalbumin-positive GABAergic interneurons. Decreased PPI and alterations of parvalbumin-positive interneurons are also endophenotypes that typically occur in schizophrenia. To verify the results found in mice, we genotyped 27 single nucleotide polymorphisms (SNPs) within the *CRYBB2* gene and its flanking regions and investigated different schizophrenia typical endophenotypes in a sample of 510 schizophrenia patients and 1322 healthy controls. In the case-control study, no association with schizophrenia was found. However, 3 of the 4 investigated haplotype blocks indicated a decreased *CRYBB2* mRNA expression. Two of these blocks were associated with poorer antisaccade task performance and altered working memory-linked functional magnetic resonance imaging signals. For the two haplotypes associated with antisaccade performance, suggestive evidence was found with visual memory and in addition, haplotype block 4 showed a nominally significant association with reduced sensorimotor gating, measured as P50 ratio. These results were not schizophrenia-specific, but could be detected in a combined sample of patients and healthy controls. This is the first study to demonstrate the importance of βB2-crystallin for antisaccade performance and memory function in humans and therefore provides implications for βB2-crystallin function in the human brain.

## Introduction

Crystallins are the main structural proteins in the ocular lens and several mutations have been described that lead to congenital cataract (for a recent review see Graw^1^). In the recent past, it has been shown that different crystallin genes are expressed in the mammalian brain. Thus, α-crystallins have been associated with neurodegenerative diseases such as Alzheimer’s disease, Parkinson’s disease and Alexander’s disease (for review see Graw^2,3^ and references therein).

βB2-crystallin (gene symbol: *CRYBB2* in humans, and *Crybb2* in mice), a member of the β/γ-crystallin superfamily, occurs not exclusively in the ocular lens, but is also expressed in various brain regions such as the hippocampus, olfactory bulb, cerebellum and cerebral cortex^4^. It has been shown that βB2-crystallin is involved in axonal regeneration^5^ and dendritogenesis^6^. Moreover, βγ-crystallins have the property of binding calcium with moderate affinity^7,8^.

In mice, three mutations (*Philly*, *Aey2*, *O377*) have been described, all of which affect the last of the 7 exons^4,9,10^. In 2013, Sun et al.^11^ first described detailed molecular, cellular, electrophysiological and behavioral alterations in βB2-crystallin deficient male *O377* mutants. Compared to wildtype mice, *O377* mutants displayed a decreased acoustic startle reflex (ASR) in combination with an increased prepulse inhibition (PPI). A recently published study confirmed alterations of sensorimotor gating (PPI) in both sexes in all 3 mouse mutant lines^12^. In addition, altered numbers of parvalbumin-positive interneurons were observed in the thalamic reticular nucleus (TRN) of these mice, which is consistent with results showing that a deficiency of parvalbumin, a calcium binding protein, is known to affect PPI^13^.

Remarkably, deficits in PPI^14–16^ and a reduced number of parvalbumin-positive interneurons in the hippocampus and TRN^17,18^ are typical findings in patients with schizophrenia. PPI is an operationalized measurement of sensorimotor gating and deficits have been observed in several neuropsychiatric disorders, being most extensively studied in schizophrenia. Reduced PPI has been described in patients with a first episode of schizophrenia as well as in unaffected relatives^19^ and is therefore an appropriate endophenotype of schizophrenia. In addition to the deficits in sensorimotor gating, *O377* mice showed a reduction of hippocampal volume, which has also been found in patients with schizophrenia^11,20^.

Based on these findings, *CRYBB2* appears to be an interesting candidate gene for schizophrenia, a complex and multifactorial psychiatric disease in which genetic factors play a major role. Numerous rare and common genetic variants associated with schizophrenia have been identified so far^21^, including a region on chromosome 22q11.2 that has long been associated with the velo-cardio-facial syndrome (VCFS), also known as DiGeorge syndrome^22^. This syndrome, which, in addition to widely varying malformations including cardiac, palatal and endocrine abnormalities, leads to schizophrenia-like psychotic symptoms and is also associated with deficits in PPI and antisaccade task performance^23–25^. In most patients, a microdeletion of about 3 Mbp can be found near the *CRYBB2* locus (22q11.23), but unusual deletions have also been described in this region. A specific gene causing DiGeorge syndrome has not yet been identified and due to phenotypic variability and different penetrance it can be assumed that the exact location and size of the microdeletion determines the manifestation^26,27^. The fact that different schizophrenia related endophenotypes are genetically influenced is confirmed by a study conducted by Greenwood et al.^28^ in which the heritability of oculomotor inhibition (antisaccades) was estimated at 42% and that of the neurocognitive endophenotypes verbal learning and working memory at 25% and 39%, respectively. Although no heritability for P50 suppression was found in this study, other studies showed that this endophenotype is also highly heritable^29,30^.

In the largest genome-wide association study (GWAS) on schizophrenia to date, however, no association was found for *CRYBB2* (Ripke et al.). Beyond schizophrenia, a meta-analysis of quantitative trait loci (eQTL) in the brain performed on a total of 424 samples from neuropathologically healthy subjects, a SNP (rs997872) in the *CRYBB2* containing chromosomal region was the most significant hit^31^ pointing to a variation specific differential expression of this protein in the brain. In addition to the findings from animal models, this result supports the potential relevance of CRYBB2 for brain function.

Here, in addition to a genetic association study in schizophrenia patients and healthy controls, we report converging evidence that polymorphisms in the *CRYBB2* gene and its flanking regions are linked to altered *CRYBB2* mRNA expression, antisaccade task performance, and working memory performance.

## Methods

### Schizophrenia patients and healthy controls

All individuals included in this exploratory study were part of the PAGES sample^32^. All schizophrenia patients were recruited from the Munich area in Germany. 66% of them were of German descent (i.e., both parents were German), 34% were other Caucasians from middle Europe. Detailed medical and psychiatric histories were collected, including the Structured Clinical Interview for DSM-IV (SCID), to evaluate lifetime axis I and II diagnoses^33,34^. All patients had a diagnosis of schizophrenia based on DSM-IV (Diagnostic and Statistical Manual of Mental Disorders) and ICD-10 criteria. Patients with a history of traumatic head injury, substance abuse before age at onset and suspected organic psychosis (due to cerebral neoplasia, stroke, hemorrhage, inflammation) were not included in the study. At the time of the investigations, the patients were stable and outside an acute psychotic phase.

The control group consisted of unrelated volunteers of self-reported German descent (i.e. both parents German) and was randomly selected from the general population of Munich, Germany, and contacted by mail. Subjects with neuropsychiatric disorders assessed with the SCID or subjects with first-degree relatives with neuropsychiatric disorders assessed with the Family History Assessment Module^35^ were excluded.

Written informed consent was obtained from all subjects after a detailed and extensive description of the study, which was approved by the ethics committee of the Ludwig-Maximilians-University Munich and carried out in accordance to the ethical standards laid down in the Declarations of Helsinki.

### Genotyping

SNPs (Single Nucleotide Polymorphisms) with a physical distance of approximately 1 kb were selected from the NCBI dBSNP database (http://www.ncbi.nlm.nih.gov/entrez/query.fcgi?db=snp) and from Pubmed publications (http://www.ncbi.nlm.nih.gov/entrez/query.fcgi?db=PubMed). The “Tagger” pairwise method (http://software.broadinstitute.org/mpg/tagger/server.html) was used to ensure all LD blocks were covered. In addition, proxies were included to avoid technical replications. Only validated SNPs with a minor allele frequency of more than 1% were selected. 27 SNPs were included covering ~25 kbp of the *CRYBB2* gene and its flanking regions (Chr22: 25216313 – 25240713; GRCh38.p12) with a mean distance of 879 bp per SNP. DNA was extracted from EDTA blood using QiaAmp DNA blood maxi kit (Qiagen, Hilden, Germany) and the concentration was adjusted using the PicoGreen quantitation reagent (Invitrogen, Karlsruhe, Germany). Afterwards, 2.5 ng DNA were genotyped using the iPLEX assay on the MassARRAY MALDI-TOF mass spectrometer (Sequenom, Hamburg, Germany). Genotyping call rates in cases and controls were above 99%. Allele frequencies were similar to CEU sample frequencies. All SNPs were in Hardy–Weinberg Equilibrium (*p* > 0.05).

### mRNA expression

Lymphocytes from whole blood were separated using a Ficoll gradient. RNA was extracted from lymphocytes using RNeasy Mini Kit (Qiagen, Hilden, Germany). 1 µg of total RNA was used for cDNA synthesis with random hexamer primers (Applied Biosystems, Darmstadt, Germany) and Superscript II (Invitrogen, Karlsruhe, Germany) according to the manufacturer’s recommendations. Quantitative real-time PCR was performed in duplicates in a Rotor-Gene 2000 Cycler (Corbett Research, Sydney, Australia). 100 ng total RNA, 12.5 µl ABsolute QPCR SYBR Green Mix (ABgene, Hamburg, Germany) and 10 pM of each primer were used in a 25 µl reaction. Expression was normalized by interrun calibration based on 10% sample overlap per run using qBase (http://medgen.ugent.be/qbase/). The ratios of the normalized expression (*CRYBB2*/*POLR2A*) values were calculated.

### P50 event-related potential assessments

P50 event-related potential assessments were carried out to assess sensorimotor gating. Therefore, an auditory click-pairs paradigm with 0.04 msec duration of square wave pulses was used. The interval between click pairs was 10 s. Three sets of averaged evoked responses to 36 pairs of stimuli were obtained from subjects at an interval of 500 msec between the conditioning stimulus (first click) and the test stimulus (second click). Auditory dysfunction was excluded by auditory testing of the hearing threshold with a Philips audiometer. Recording took place in a sound-attenuated and electrically shielded room adjacent to the recording apparatus (Neuroscan Synamps, Hamburg, Germany). Electrodes were positioned according to the 10/20 system with the additional electrodes FC1, FC2, FC5, FC6, CP5, CP6, PO9, and PO10. Data were collected with a sampling rate of 1000 Hz and a bandpass filter of 0.16–200 Hz. The preprocessing of the EEG was done with the Vision Analyzer software Vers. 1.05 (Brain Products, Gilching, Germany). Grand averages were calculated for each individual. Voltage changes exceeding more than 70 µV were excluded. Only wave-shapes based on at least 70 segments (from 108) were accepted. Mean numbers of individual trial segments were 88.9 (8.8) for healthy subjects and 88.1 (9.7) for patients with schizophrenia. Accordingly, there was no significant difference concerning the number of trials used in the grand averages between the groups (*F* = 0.27, *p* = 0.6). For the first click, the software selected the most positive peak between 30 and 80 msec after the conditioning stimulus. Amplitudes were measured relative to the previous negativity. The test response was selected (by visual inspection) within a window (±10 msec) of the conditioning stimulus response latency. P50 ratios were calculated as test stimulus response/conditioning stimulus response (T/C ratio) and used for further analyses. Only data-sets with detectable test response and ratios below two (with typical ratios below one) were included.

### Antisaccade task

Eye movement recordings were performed as previously described^36^. The following anti-gap protocol was used: 50 ms before the target appeared, the fixation light was switched off. The target then appeared at random positions 5 or 15 deg to the left or to the right of the fixation spot. The subjects were instructed to make a saccade that is opposite to where the target appeared (antisaccade). Percentage of initial saccades opposite (performance) and towards the cue (errors) were the variables used for further analyses.

### Memory measurement via the Wechsler memory test

The Wechsler Memory Scale-Revised (WMS-R) assesses verbal and nonverbal memory ability in adults^37^. It was developed to examine learning, memory and attention using both auditory and visual stimuli. The manual provides 13 subtests on different tasks including verbal memory, visual memory, general memory, attention and delayed memory. The subtests “Information and Orientation” and “Mental Control” were not included in the analyses.

### Functional magnetic resonance imaging (fMRI) and working memory tasks

In a subset of participants, fMRI data were acquired using a 1,5 T scanner (Sonata, Siemens, Erlangen, Germany). During the scan, the subjects had to perform the N-back task to test working memory performance (for detailed information see Callicot^38^ and Callicot et al.^39^). Image processing and statistical analyses were carried out using the Brainvoyager software package (Version 4.9; Brain Innovation, Maastricht, Netherlands). The five images at the beginning of each session were deleted due to inhomogeneities of the magnetic field. Movement was less than 3 mm or 3 degrees rotation in any direction in all scans with all subjects. The following post-processing procedures were conducted: high-pass filter, slice timing, correction of motion artifacts, smoothing (8-mm Gaussian kernel), and the images were transformed to a standard Talairach brain. First, level analyses were performed on each subject, estimating the variance of voxels according to a general linear model. Each task condition was modeled as a boxcar function and convolved with the hemodynamic response function. Regions of interest (ROIs) were defined according to the results on healthy control subjects presented in Karch et al.^40^. ROIs were calculated taking into account the increase of hemodynamic response during the 2-back condition compared to the 0-back task thresholded at *p* < .00001 (*T* ≥ 4.8; random effects analysis) for the control group. ROIs were defined for regions in the left and right orbitofrontal, lateral and medial prefrontal and parietal cortex, respectively. The number of activated voxels during the task conditions within these ROIs was calculated separately for each participant (*p* < .005 uncorrected for multiple comparisons).

### Further statistical analyses

Haploview 3.2 (https://www.broadinstitute.org/haploview/haploview)^41^ was used to generate a linkage disequilibrium (LD) map (D’) and to test for deviation from Hardy-Weinberg equilibrium (HWE) at a significance level of *p* < 0.05. Tests for differences between cases and controls for alleles and genotypes in single SNPs were performed using the Chi-square test. Tests for associations of quantitative traits (*CRYBB2* mRNA expression, P50 ratio, correct responses in antisaccade task) with multimarker haplotypes were performed using the software package “haplo.score”^42^ of the software environment “R” (www.r-project.org). Simulated p-values were determined by recording of the trait and covariates keeping the original genotype matrix over 10,000 replicates (simulated p-value; sim.p). Covariates sex, age, education and diagnosis were integrated as appropriate. Association analyses of memory function (WMS-R) and risk haplotypes were performed by SPSS using multivariate analysis of covariance (MANCOVA). Age and education were added as covariates, sex was added as fixed factor. Affection status and the interaction of haploblock allele and affection status were additionally added for the combined sample containing cases and controls. Functional MRI association analyses of risk haplotypes were calculated using ANOVA including age, sex and education as covariates. Endophenotype associations were determined for haplotypes indicating altered mRNA *CRYBB2* expression (*p* < 0.1). Correction for multiple testing was performed separately for each endophenotype using Bonferroni adjusted significance levels correcting for the number of conducted tests as follows: antisaccade task performance (correct and incorrect antisaccades * number of haploblocks; *p* < 8.3 × 10^−3^), P50 ratio (number of haploblocks; *p* < 1.7 × 10^−2^), memory performance measured with the WMS-R (number of subtests * haploblocks; *p* < 1.5 × 10^−3^) and fMRI (4 main brain regions of both hemispheres * number of haploblocks; *p* < 2.1 × 10^−3^). A *p*-value < 0.05 was considered nominally significant.

## Results

### Sample description

1832 individuals of Caucasian descent participated in this study. The schizophrenia group consisted of 334 males and 176 females with a mean age of 38.9 years (SD 10.3 years) and has already been described^43–45^. Based on DSM-IV and ICD-10 diagnosis, patients could be allocated to the following subgroups: paranoid 77.6%, disorganized 15.6%, catatonic 2.2% and undifferentiated 4.6%. Among the 510 patients, 450 received antipsychotic recurrence prophylaxis with one or more drugs, while 135 patients received typical and 383 patients received atypical neuroleptics and 35 patients received mood stabilizers (e.g., valproate).

The control group consisted of 606 male und 716 female healthy volunteers with a mean age of 48.1 years (SD 14.8 years). Studies on mRNA expression, sensorimotor gating, antisaccade task performance and memory performance were conducted in subsets of this sample, which is described in detail in the corresponding section.

### Genetic association study

**Fig. 1:**
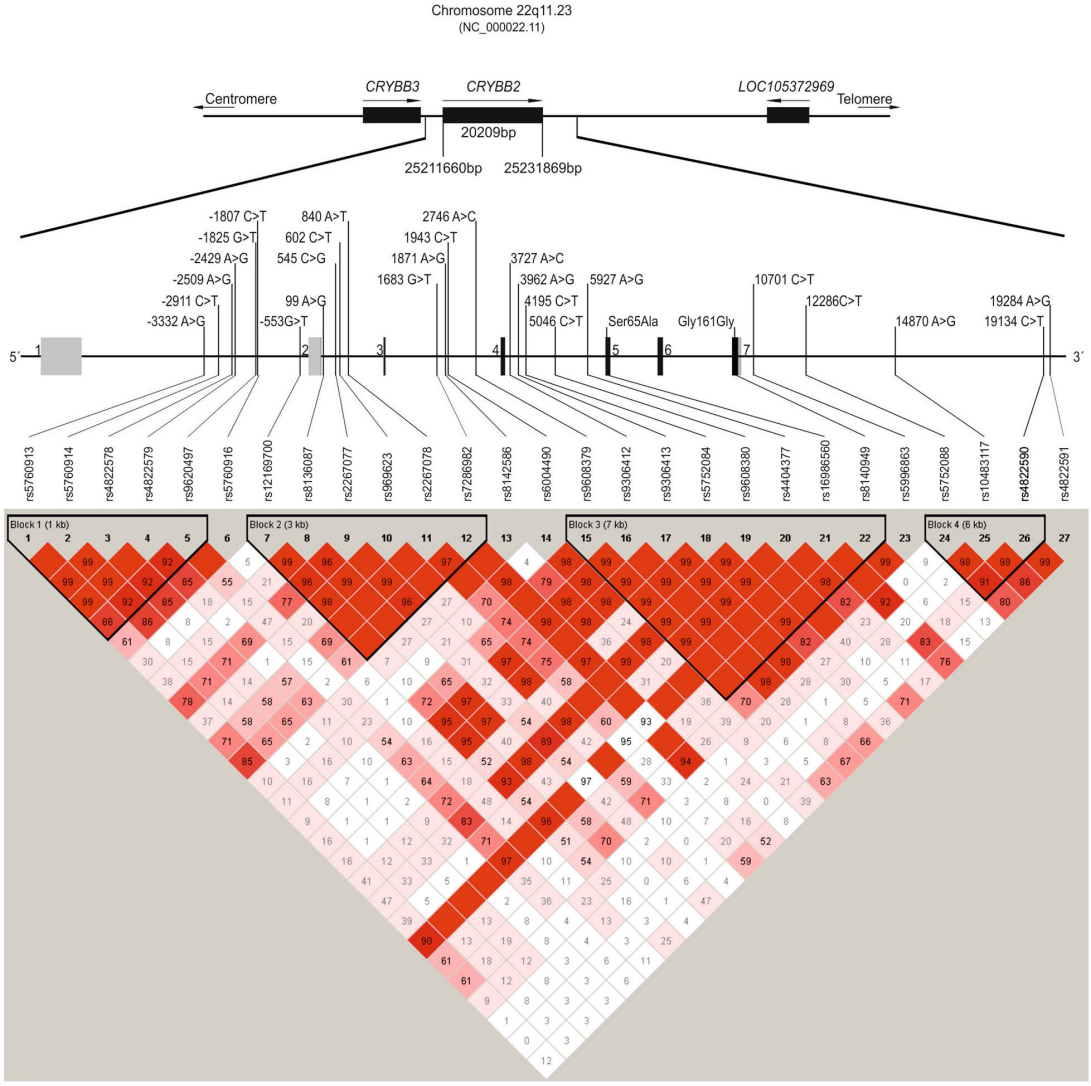
CRYBB2 gene region and investigated SNPs. Overview of the *CRYBB2* gene region, *CRYBB2* intron/exon structure and localization of the 27 investigated SNPs. Linkage Disequilibrium (LD) of the 27 SNPs is presented based on the genotypes of the combined group. Regions of high LD (*D*’ = 1 and LOD > 2) are shown in bright red; decreasing color intensity indicates decreasing *D*’-value. Regions of low LD and low LOD scores (*D*’ < 0.2, LOD < 2) are shown in white.

The *CRYBB2* gene is located on chromosome 22q11.23. 27 SNPs within a ~25 kbp segment covering the *CRYBB2* gene and its flanking regions (Chr22: 25216313 - 25240714; GRCh38.p12) were investigated and four haplotype blocks have been detected (Fig. 1). Although the *CRYBB2* gene is located in the extended region of the 22q11.2 deletion syndrome, which is responsible for VCFS syndrome and has been repeatedly linked to psychotic symptoms^22^, we found no association with schizophrenia, neither in the analysis of individual SNPs nor of the four haplotype blocks.

### mRNA expression

Measurements of *CRYBB2* mRNA expression were performed using lymphocytes from 89 subjects (46 patients and 43 healthy controls). Carriers of the GAGTAG haplotype of the 2^nd^ block and carriers of CAACCGGG haplotype of the 3^rd^ block were observed to have a significantly lower expression of *CRYBB2* mRNA. A trend towards lower expression was observed for TAC carriers in block 4 (Table 1b, d, f). No significant differences were found between patients and controls (data not shown). Those haplotype blocks suggesting a functional consequence by association with a lower *CRYBB2* mRNA expression (*p* < 0.1) were taken into consideration for further analyses.

**Table 1:** Association analyses of haplotype blocks.

Haplotype	Frequency %	HaploScore	sim. *p*-value
**a: haplotype block 2: antisaccade eye movement**			
**GAGTAG**	**8.6**	−**3.103**	**1.0** × **10**^−**3#**^
GACTTT	52.5	−0.671	5.1 × 10^−1^
TACTTT	7.0	0.183	8.6 × 10^−1^
TGCCTT	21.3	1.347	1.8 × 10^−1^
GAGTAT	1.6	1.370	1.8 × 10^−1^
GAGTTT	8.0	1.441	1.5 × 10^−1^
**b: haplotype block 2: mRNA expression**			
**GAGTAG**	**7.4**	−**2.029**	**4.1** × **10**^−**2**^
GAGTAT	1.1	−1.083	3.0 × 10^−1^
TGCCTT	25.3	−0.449	6.5 × 10^−1^
TACTTT	5.7	−0.204	8.4 × 10^−1^
GAGTTT	8.6	0.299	7.6 × 10^−1^
GACTTT	51.7	1.629	9.7 × 10^−2^
**c: haplotype block 3: antisaccade eye movement**			
**CAACCGGG**	**9.0**	−**2.911**	**4.0** × **10**^−**3 #**^
ACGTCGGG	3.7	−1.241	2.1 × 10^−1^
CAACTGGG	16.3	−0.131	9.0 × 10^−1^
ACGTCAGA	20.5	0.193	8.5 × 10^−1^
ACGTCATA	2.3	0.211	8.3 × 10^−1^
ACGCCGGG	23.0	0.636	5.3 × 10^−1^
ACGTCAGG	23.5	1.247	2.1 × 10^−1^
CCGCCGGG	1.7	1.822	6.8 × 10^−1^
**d: haplotype block 3: mRNA expression**			
**CAACCGGG**	**7.1**	−**2.068**	**3.5** × **10**^−**2**^
CCGCCGGG	2.3	−1.281	2.1 × 10^−1^
ACGTCATA	2.6	−0.685	5.0 × 10^−1^
ACGCCGGG	26.6	−0.554	5.8 × 10^−1^
ACGTCAGA	21.2	0.608	5.4 × 10^−1^
ACGTCGGG	4.2	0.856	4.0 × 10^−1^
CAACTGGG	15.4	0.864	4.0 × 10^−1^
ACGTCAGG	20.5	0.921	3.6 × 10^−1^
**e: haplotype block 4: P50 T/C Ratio**			
**TAC**	**36.6**	−**2.039**	**4.6** × **10**^−**2**^
CAC	17.8	0.129	9.0 × 10^−1^
CGC	19.7	0.767	4.5 × 10^−1^
CAT	0.8	0.830	4.2 × 10^−1^
TAT	24.7	1.546	1.2 × 10^−1^
**f: haplotype block 4: mRNA expression**			
TAC	35.5	−1.773	7.4 × 10^−2^
TGC	0.6	0.179	8.0 × 10^−1^
CGC	26.3	0.490	6.3 × 10^−1^
TAT	23.6	0.695	4.9 × 10^−1^
CAC	13.9	0.912	3.7 × 10^−1^

**a**, **b** Association analysis of haplotype block 2 with antisaccade task performance (correct responses) and mRNA expression of *CRYBB2* from lymphocytes. **c**, **d** Association analysis of haplotype block 3 with antisaccade task performance (correct responses) and mRNA expression of *CRYBB2* from lymphocytes. **e**, **f** Association analysis of haplotype block 4 with sensorimotor gating measured as P50 ratio and mRNA expression of *CRYBB2* from lymphocytes.

Nominal significant *p*-values (*p* < 0.05) are marked bold, ^#^*p*-value passes Bonferroni correction for number of endophenotype-specific analyses performed and number of haploblocks.

### Sensory gating and antisaccades

As alterations of the PPI were also found in *Crybb2* mutant mice^12^, we studied the association between genetic polymorphisms in the *CRYBB2* gene and sensorimotor gating in humans. A subset of 352 subjects (109 patients, 243 healthy volunteers) participated in P50 event-related potential assessments. We observed a suggestive association of a lower P50 ratio (T/C) with the TAC-haplotype in block 4 downstream of the last exon (ratio T/C: carrier: 0.64 (0.71); non-carrier: 0.71 (0.44); *p* = 0.046; Table 1e).

We also performed an antisaccade task in 223 participants (81 patients and 142 healthy volunteers). Carriers of the GAGTAG haplotype of the 2^nd^ block and carriers of the CAACCGGG haplotype of the 3^rd^ block showed poorer performance in antisaccade task, measured as percentage of correct responses (block 2: *p* = 0.001; carrier: 36.0% (25.1); non-carrier: 45.6% (27.5); block 3: *p* = 0.004; carrier: 35.4% (25.1); non-carrier: 45.6% (27.5); Table 1a, c). After correction for multiple testing for the number of analyses performed and the number of haplotype blocks investigated, the association of risk haplotypes of blocks 2 and 3 remained significant.

### Memory

Based on the reduced hippocampal size of *Crybb2* mutant mice and the crucial role of the hippocampus in memory formation, we performed tasks on short- and long-term memory as well as on working memory measured with the WMS–R^37^. A total of 538 participants took part, including 181 schizophrenia patients and 357 healthy controls. Furthermore, we complemented these investigations with an fMRI-based measurement of working memory using the N-back task in 174 participants, including 53 patients and 121 healthy subjects.

Short- and long-term memory performance was measured using the WMS-R and tested against haplotypes implicated in altered *CRYBB2* mRNA expression (Table 2). In the patient group, nominally significant differences with better performance of GAGTAG carriers of block 2 and CAACCGGG in block 3 were observed for immediate and delayed visual reproduction. In addition, a nominally significant interaction effect was detected, but with poorer performance in immediate and delayed visual reproduction for healthy risk haplotype carriers. There were also suggestive significant results for block 4, with TAC carriers showing worse results for immediate and delayed verbal and visual paired associates in the combined sample. These exploratory results should be treated with caution due to multiple testing.

**Table 2:** Association of haplotypes with subscales of the WMS-R.

	Haploblock 2 GAGTAG vs. all other				Haploblock 3 CAACCGGG vs. all other				Haploblock 4 TAC vs. all other			
	All	H × G	Controls	Cases	All	H × G	Controls	Cases	All	H × G	Controls	Cases
**Attention**												
***Immediate***												
Digit-Span	7.4 × 10^−1^	1.4 × 10^−1^	4.3 × 10^−1^	2.7 × 10^−1^	7.1 × 10^−1^	1.7 × 10^−1^	5.0 × 10^−1^	3.2 × 10^−1^	4.3 × 10^−1^	6.4 × 10^−1^	7.4 × 10^−1^	4.5 × 10^−1^
Spatial-Span	7.5 × 10^−1^	2.7 × 10^−1^	5.4 × 10^−1^	4.0 × 10^−1^	6.3 × 10^−1^	2.3 × 10^−1^	5.7 × 10^−1^	3.2 × 10^−1^	9.0 × 10^−1^	8.0 × 10^−1^	7.5 × 10^−1^	9.7 × 10^−1^
**Verbal memory**												
***Immediate***												
Logical memory	7.6 × 10^−1^	7.5 × 10^−1^	3.9 × 10^−1^	9.9 × 10^−1^	9.9 × 10^−1^	6.5 × 10^−1^	5.1 × 10^−1^	8.0 × 10^−1^	7.6 × 10^−1^	8.2 × 10^−1^	8.8 × 10^−1^	8.7 × 10^−1^
Verbal paired Associates	1.9 × 10^−1^	3.3 × 10^−1^	7.7 × 10^−1^	2.3 × 10^−1^	1.6 × 10^−2^	3.7 × 10^−1^	4.1 × 10^−1^	2.3 × 10^−1^	**2.8** × **10**^**−2**^	5.4 × 10^−2^	8.0 × 10^−1^	7.1 × 10^−2^
***Delayed***												
Logical memory	9.5 × 10^−1^	3.1 × 10^−1^	4.1 × 10^−1^	9.9 × 10^−1^	8.0 × 10^−1^	3.2 × × 10^−1^	3.1 × 10^−1^	7.5 × 10^−1^	5.6 × 10^−1^	4.0 × 10^−1^	7.2 × 10^−1^	4.4 × 10^−1^
Verbal paired Associates	3.0 × 10^−1^	2.3 × 10^−1^	9.0 × 10^−1^	1.8 × 10^−1^	2.1 × 10^−1^	1.9 × 10^−1^	8.3 × 10^−1^	1.5 × 10^−1^	**3.7** × **10**^**−2**^	2.9 × 10^−1^	9.5 × 10^−1^	1.4 × 10^−1^
**Visual memory**												
***Immediate***												
Figural memory	6.1 × 10^−2^	1.2 × 10^−1^	7.5 × 10^−1^	7.9 × 10^−2^	9.6 × 10^−2^	3.5 × 10^−1^	5.0 × 10^−1^	2.1 × 10^−1^	8.0 × 10^−2^	5.4 × 10^−1^	**2.0** × **10**^**−2**^	5.9 × 10^−1^
Visual paired Associates	4.6 × 10^−1^	5.2 × 10^−1^	7.7 × 10^−1^	3.9 × 10^−1^	5.2 × 10^−1^	5.8 × 10^−1^	4.1 × 10^−1^	4.9 × 10^−1^	**2.4** × **10**^**−2**^	1.2 × 10^−1^	5.2 × 10^−1^	7.7 × 10^−2^
Visual reproduction	3.5 × 10^−1^	**2.6** × **10**^**−3**^	7.9 × 10^−2^	**4.0** × **10**^**−2**^	3.1 × 10^−1^	**1.7** × **10**^**−3**^	6.8 × 10^−2^	**4.5** × **10**^**−2**^	2.4 × 10^−1^	7.0 × 10^−1^	8.3 × 10^−2^	5.3 × 10^−1^
***Delayed***												
Visual paired associates	3.1 × 10^−1^	3.0 × 10^−1^	7.4 × 10^−1^	2.5 × 10^−1^	2.0 × 10^−1^	2.1 × 10^−1^	7.1 × 10^−1^	1.9 × 10^−1^	**2.3** × **10**^**−2**^	**2.1** × **10**^**−2**^	9.5 × 10^−1^	**5.0** × **10**^**−2**^
Visual reproduction	3.1 × 10^−1^	**6.1** × **10**^**−3**^	1.8 × 10^−1^	**4.4** × **10**^**−2**^	2.3 × 10^−1^	**3.4** × **10**^**−3**^	1.8 × 10^−1^	**3.8** × **10**^**−2**^	5.1 × 10^−1^	7.0 × 10^−1^	2.4 × 10^−1^	6.5 × 10^−1^

H × G = interaction effect of haplotype and group; significant *p*-values in bold.

**Fig. 2:**
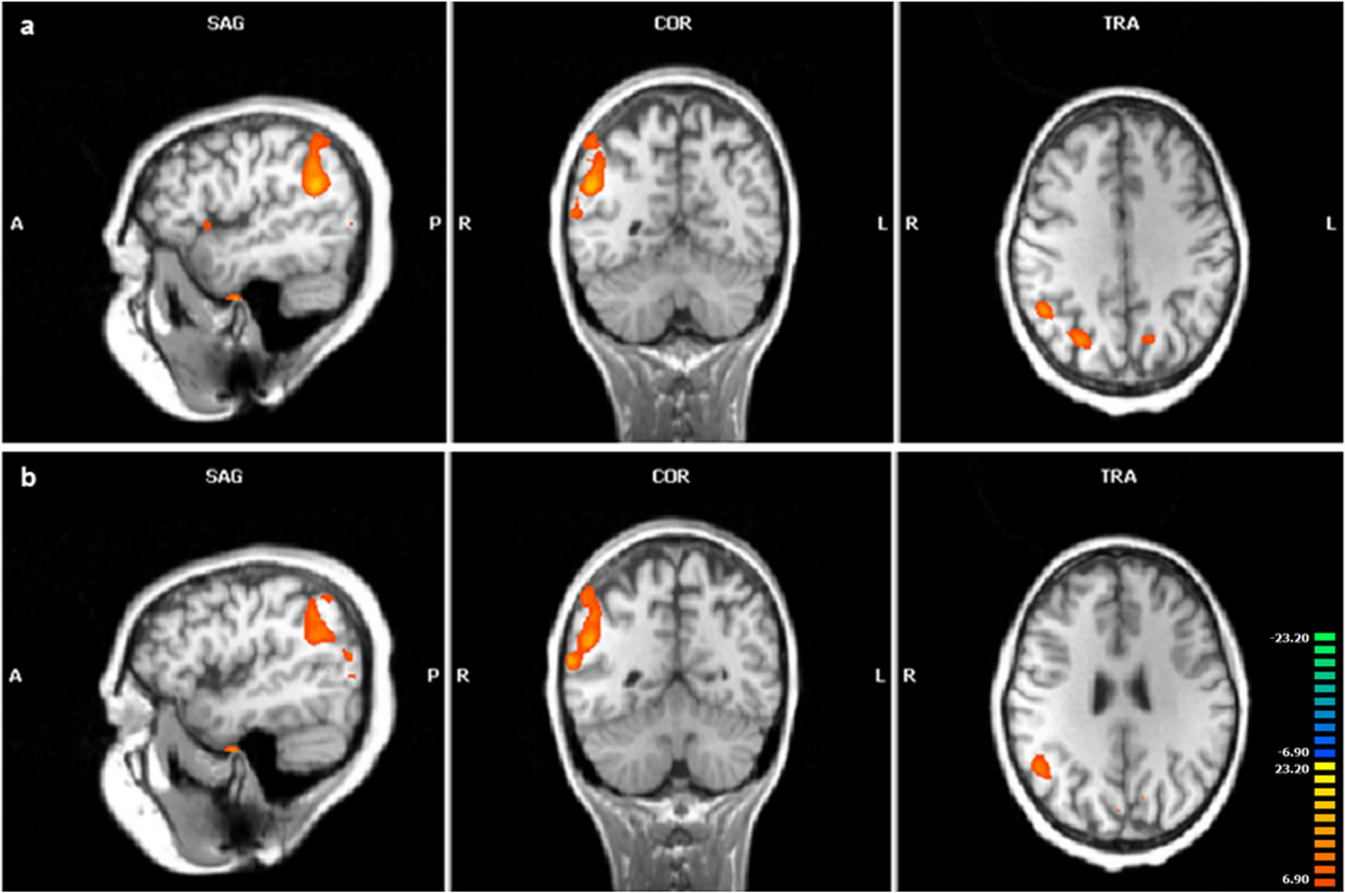
Functional MRI. Functional MRI results during a working memory task (2- minus 0-back) are demonstrated for sagittal (SAG), coronal (COR) and transversal (TRA) sections. (a: anterior; p: posterior; r: right; l: left). ANCOVA was calculated including haplotype status, age, sex and education as covariates (*T*-score: 6.9–23.2; *p*_(uncor)_ <0.01). **a** Haploblock 2: GAGTAG carriers were analyzed vs. non-carriers (talairach coordinates: *x* = 44; *y* = −53; *z* = 34). **b** Haploblock 3: CAACCGGG carriers were analyzed vs. non-carriers (talairach coordinates: *x* = 44; *y* = −50; *z* = 28).

In addition, we investigated whether there is a connection between the three different risk haplotypes and working memory-connected fMRI signals. The main results are presented in Table 3. Interestingly, almost all ROIs measured in the prefrontal or parietal region (right hemisphere) were associated with the GAGTAG haplotype of block 2 and the CAACCGGG haplotype of block 3 (Fig. 2a, b). Carriers of these two haplotypes showed a higher activation during the fMRI task suggesting ineffective activation compared to non-carriers. In detail, significant associations after Bonferroni correction were found for the risk haplotype of block 2 with higher activation in the right orbitofrontal, prefrontal and parietal brain regions in the combined sample. The direction of association of increased activation in the right orbitofrontal and prefrontal regions with the presence of the block 2 risk haplotype (representing a potentially lower mRNA expression) was indicated by nominal significance in both subgroups, controls as well as schizophrenia cases. The associations of the right parietal region with the risk haplotype of block 2 remained significant in the control group only. In addition, the right parietal region was significantly associated with the risk haplotype of block 3 in the combined group. No association with the TAC haplotype of block 4 could be detected.

**Table 3:** Functional MRI results during a working memory task (2- minus 0-back); the respective *p*-values are given (significant *p*-values bold marked).

2-back minus 0-back			Haploblock 2 GAGTAGvs. all other			Haploblock 3 CAACCGGGvs. all other			Haploblock 4 TACvs. all other		
	Center of gravity	Brodman Area	Controls	Cases	All	Controls	Cases	All	Controls	Cases	All
	Talairach x, y, z										
**Left**											
**Orbitofrontal**	−37, 18, 13	13	1.4 × 10^−1^	1.9 × 10^−1^	5.9 × 10^−2^	2.4 × 10^−1^	1.9 × 10^−1^	1.0 × 10^−1^	5.5 × 10^−1^	3.6 × 10^−1^	7.8 × 10^−1^
**Mediofrontal**	−6, 10, 54	6	3.3 × 10^−1^	2.9 × 10^−1^	2.1 × 10^−1^	3.8 × 10^−1^	2.9 × 10^−1^	2.5 × 10^−1^	4.7 × 10^−1^	6.6 × 10^−1^	7.6 × 10^−1^
**Prefrontal (total)**			3.9 × 10^−1^	3.3 × 10^−1^	3.1 × 10^−1^	4.3 × 10^−1^	3.3 × 10^−1^	3.5 × 10^−1^	9.2 × 10^−1^	2.3 × 10^−1^	3.8 × 10^−1^
Prefrontal (1)	−34, 46, 23	10	7.8 × 10^−1^	2.0 × 10^−1^	6.0 × 10^−1^	8.4 × 10^−1^	2.0 × 10^−1^	6.8 × 10^−1^	2.7 × 10^−1^	5.1 × 10^−1^	2.2 × 10^−1^
Prefrontal (2)	−38, 14, 37	9	4.6 × 10^−1^	5.2 × 10^−1^	4.0 × 10^−1^	5.0 × 10^−1^	5.2 × 10^−1^	4.5 × 10^−1^	8.4 × 10^−1^	1.8 × 10^−1^	4.5 × 10^−1^
Prefrontal (3)	−21, 0, 59	6	9.7 × 10^−2^	2.2 × 10^−1^	6.0 × 10^−2^	1.1 × 10^−1^	2.2 × 10^−1^	6.9 × 10^−2^	5.6 × 10^−1^	3.8 × 10^−1^	9.0 × 10^−1^
**Parietal (total)**			8.3 × 10^−2^	4.8 × 10^−1^	7.9 × 10^−2^	9.5 × 10^−2^	4.8 × 10^−1^	9.3 × 10^−2^	8.7 × 10^−1^	7.7 × 10^−1^	7.9 × 10^−1^
Parietal (1)	−38, −54, 41	40	1.1 × 10^−1^	7.5 × 10^−1^	1.5 × 10^−1^	1.2 × 10^−1^	7.5 × 10^−1^	1.6 × 10^−2^	8.1 × 10^−1^	8.1 × 10^−1^	7.6 × 10^−1^
Parietal (2)	−9, −69, 47	7	9.2 × 10^−2^	**3.0** × **10**^**−2**^	**1.4** × **10**^**−2**^	1.2 × 10^−1^	**3.0** × **10**^**−2**^	**2.0** × **10**^**−2**^	8.6 × 10^−1^	6.9 × 10^−1^	1.0
**Right**											
**Orbitofrontal**	40, 22, 9	13	**1.4** × **10**^**−2**^	**1.5** × **10**^**−2**^	**6.2** × **10**^**−4 #**^	**5.0** × **10**^**−2**^	**1.5** × **10**^**−2**^	**3.3** × **10**^**−3**^	3.6 × 10^−1^	5.4 × 10^−1^	7.7 × 10^−1^
**Mediofrontal**	9, 18, 48	8	8.0 × 10^−2^	1.0 × 10^−1^	**2.6** × **10**^**−2**^	1.6 × 10^−1^	1.0 × 10^−1^	6.1 × 10^−2^	8.4 × 10^−1^	8.7 × 10^−1^	1.0
**Prefrontal (total)**			**3.4** × **10**^**−2**^	**6.8** × **10**^**−3**^	**1.3** × **10**^**−3 #**^	6.8 × 10^−2^	**6.8** × **10**^**−3**^	**3.4** × **10**^**−3**^	8.9 × 10^−1^	9.0 × 10^−1^	8.8 × 10^−1^
Prefrontal (1)	34, 47, 25	10	1.8 × 10^−1^	**1.3** × **10**^**−2**^	**1.5** × **10**^**−2**^	2.7 × 10^−1^	**1.3** × **10**^**−2**^	**2.9** × **10**^**−2**^	9.2 × 10^−1^	5.4 × 10^−1^	7.4 × 10^−1^
Prefrontal (2)	35, 26, 42	8	9.0 × 10^−2^	**3.3** × **10**^**−2**^	**9.9** × **10**^**−3**^	1.3 × 10^−1^	**3.3** × **10**^**−2**^	**1.7** × **10**^**−2**^	6.6 × 10^−1^	2.3 × 10^−1^	6.1 × 10^−1^
Prefrontal (3)	46, 8, 38	9	**1.2** × **10**^**−2**^	6.4 × 10^−2^	**1.5** × **10**^**−2**^	**3.1** × **10**^**−2**^	6.4 × 10^−2^	**4.5** × **10**^**−3**^	4.2 × 10^−1^	9.8 × 10^−1^	6.4 × 10^−1^
Prefrontal (4)	26, 2, 59	6	**4.0** × **10**^**−2**^	**3.1** × **10**^**−2**^	**5.1** × **10**^**−3**^	7.6 × 10^−2^	**3.1** × **10**^**−2**^	**1.2** × **10**^**−2**^	8.1 × 10^−1^	6.7 × 10^−1^	6.8 × 10^−1^
**Parietal (total)**			**1.3** × **10**^**−3 #**^	5.3 × 10^−2^	**2.1** × **10**^**−4 #**^	**3.1** × **10**^**−3**^	5.3 × 10^−2^	**5.7** × **10**^**−4 #**^	5.8 × 10^−1^	1.7 × 10^−1^	2.4 × 10^−1^
Parietal (1)	39, −56, 40	40	**1.5** × **10**^**−3 #**^	5.5 × 10^−2^	**3.1** × **10**^**−4 #**^	**3.9** × **10**^**−3**^	5.5 × 10^−2^	**8.7** × **10**^**−4 #**^	5.1 × 10^−1^	1.8 × 10^−1^	2.1 × 10^−1^
Parietal (2)	10, −69, 48	7	**8.7** × **10**^**−3**^	1.3 × 10^−1^	**1.5** × **10**^**−3 #**^	**1.4** × **10**^**−2**^	1.3 × 10^−1^	**2.6** × **10**^**−3**^	1.0	2.1 × 10^−1^	4.9 × 10^−1^

Nominal significant *p*-values (*p* < 0.05) are marked bold, ^#^*p*-value passes Bonferroni correction for number of endophenotype-specific analyses performed and number of haploblock.

## Discussion

The main role of βB2-crystallin was attributed to the ocular lens, further roles of this gene are expected as it is also expressed in the brain^4^. Although schizophrenia related endophenotypes have been observed in animal *Crybb2* mutants, no associations of genetic variants with a diagnosis of schizophrenia were found in this study. However, regardless of the affection status of the participants, *CRYBB2* haplotypes were associated with altered *CRYBB2* mRNA expression and the endophenotypes antisaccade performance, memory and working memory function and sensorimotor gating.

In this study we were able to show that two haplotypes (GAGTAG in haploblock 2 and CAACCGGG in haploblock 3) were associated with reduced *CRYBB2* mRNA expression in lymphocytes, while one haplotype (TAC in haploblock 4) showed a trend (*p* < 0.1), which is why these three haplotype blocks were selected for further analysis. The results suggest that genetic variants of *CRYBB2* seem to lead to differential expression of the gene itself. Furthermore, tissue specific regulation of *CRYBB2* expression has been demonstrated, e. g. high expression in brain tissue and low expression in lymphocytes^46^. It can be speculated that a variation specific effect may be even more pronounced in the tissue with higher expression implicating a functional relevance of the described haplotypes in the brain. In addition, another genetic variant in the *CRYBB2* region was the genome-wide top hit in an eQTL meta-analysis of gene expression in healthy brain samples^31^. Although the functional consequences of those variations point to an influence on *CRYBB2* mRNA expression levels, both regions appear to be independent (LD: *r*^2^ < 0.1). Whether there is a link between the results obtained in this study and the downstream variant of *CRYBB2*, which is associated with gene expression in the brain, remains to be determined.

For further analyses we focused on the three haplotypes showing a suggestively altered *CRYBB2* mRNA expression and thus indicate a functional consequence. A nominal association between a specific haplotype block (TAC in haploblock 4) located downstream of exon 7 of the *CRYBB2* gene with a lower P50 ratio in the overall sample was observed. Although sensorimotor gating alterations are typical symptoms in schizophrenia patients^47^ and in patients with schizotypal personality disorder, which is believed to belong to the schizophrenia spectrum^48^, our findings reveal no schizophrenia-specific effects, but were found in the combined sample. The results converge with those observed in mice in which alterations of sensorimotor gating, measured as PPI, were the most consistent behavioral findings in the three homozygous mouse mutants^12^. PPI is largely regulated by neuronal connections between the limbic cortex (including the entorhinal cortex, hippocampus and amygdala), ventral striatum, ventral pallidum and the pontine tegmentum^49,50^. Specifically, PPI is controlled by GABAergic projections from the Globus pallidus to the pedunculopontine nucleus, and PPI alterations are associated with dysfunctional GABAergic interneurons^51–53^. In a previous study, increased hippocampal output was discovered in *O377* mutants as determined by electrophysiological measurements. This may result in enhanced accumbal GABAergic activation, leading to increased inhibition of GABAergic neurons of the Globus pallidus, thus disinhibiting the pedunculopontine nucleus and ultimately increasing PPI^11^. In line with these data, it is tempting to speculate that polymorphic sites in *CRYBB2* may be involved in the molecular pathway(s) by which *CRYBB2* function affects schizophrenia-related endophenotypes. Another explanation could be different calcium binding affinities of the resulting βB2-crystallin proteins. In the TRN, which is linked to sensorimotor gating, cognition and attention^54^, an elevated number of parvalbumin-positive cells accompanied with an increase of PPI was observed in *O377* and *Philly* mouse mutants, while *Aey2* mutants exhibited an opposite effect with a lower amount of parvalbumin-positive cells and decreased PPI^12^. These effects could result from different calcium buffer capacities of the βB2-crystallin proteins, which in turn could lead to a compensating counter regulation of parvalbumin, another calcium binding protein in the brain^55^. The loss of calcium buffer activity of *O377* and *Philly* would thus lead to an upregulation of parvalbumin which would explain the increased number of parvalbumin positive-cells in these mice^12^. However, since the evidence for P50 is weak and does not survive the correction for multiple testing, the result should be interpreted with caution.

In the overall sample, we observed a significant association of the two haplotypes of the blocks 2 and 3 (GAGTAG in block 2 and CAACCGGG in block 3) with poorer antisaccade performance. In addition to reduced P50 suppression, impaired antisaccade eye movements are a schizophrenia-related intermediate phenotype (for review see Greenwood et al.^56^), both being linked to chromosome 22q11.2, a region in which the human *CRYBB2* gene is located^23–25^. Although *CRYBB2* is located outside the region most frequently affected by the 3 Mbp microdeletion, unusual deletions are described^26^. But beyond disease associated aspects, the genetic variants of *CRYBB2* may contribute to a normally distributed phenotypic variability of the investigated endophenotypes. Although it is currently unknown how these effects are mediated, in addition to the above-mentioned consequences resulting from altered calcium buffer capacities of the corresponding proteins, differences in gene expression may contribute to the varying manifestations within the endophenotypes.

Another relevant finding from *O377* mouse mutants was a reduced volume of the hippocampus, which was explained by an early specific loss of parvalbumin-positive interneurons during the first 3 months after birth, while other (inter)neuronal populations were not affected. Differences in apoptosis were not observed in adult mice, and other possible mechanisms (e.g., decreased neurogenesis or impaired neuronal maturation) were not supported by the data obtained in the mouse model^11^. The authors also discussed an altered calcium binding affinity of mutated βB2-crystallin as a possible cause for this. Since the hippocampus plays a crucial role in memory^57^, the reduced hippocampal volume in *Crybb2* mutants prompted us to study memory function in association with *CRYBB2* variants in humans, especially since it is known that schizophrenia patients show poorer memory performance and a reduction of hippocampal volume^20,58^.

The analysis of short- and long-term visual memory revealed suggestive significant associations with *CRYBB2* haplotypes. An opposite effect was observed for the haploblocks 2 and 3 in the two subsamples, with worse results for risk allele carriers in healthy controls and better results for risk allele carriers in the patient group. On the one hand, these results indicate that the allele status has different effects depending on the affection status, on the other hand, the results should be interpreted with caution due to multiple testing. However, we do not have an explanation as to why the risk allele carriers in the patient group, unlike in healthy controls, performed better, so that replication is mandatory.

Given the association of short-term memory with these haplotypes, we finally attempted to investigate the underlying physiological processes that are at least not primarily controlled by the hippocampus. For this purpose, an fMRI working memory task (N-back task) was performed, in which we demonstrated a significantly higher activation within the group of carriers of the risk haplotypes of block 2 and 3, which was replicated in both, patients and healthy controls, with highly significant results in the combined group. Higher fMRI activation was observed mainly in the right hemisphere in carriers of these haplotypes (GAGTAG in block 2 and CAACCGGG in block 3), most prominent in orbitofrontal, prefrontal and parietal regions. At least the lateralization of spatial working memory to the right hemisphere was previously discussed in connection with the complexity of the tasks to be performed and the required executive functions^59,60^. Interestingly, antisaccade performance correlates positively with working memory capacity^61^ and correct antisaccade trials induce a higher right hemispheric dorsolateral prefrontal cortex activation^62^, one of the brain regions that was affected in the fMRI working memory task in this study. Even if not all of the results obtained in this study survive multiple testing correction, the findings appear biologically plausible and suggest a possible connection that could be based on a shared functional mechanism, which might be influenced by genetic variations of the *CRYBB2* gene.

In summary, the presented data indicate associations of genetic variations in *CRYBB2* with disturbed *CRYBB2* mRNA expression, antisaccade performance, working memory and visual memory performance and sensorimotor gating, demonstrating at least partially overlapping functional aspects of βB2-crystallin in humans and mice^11,12^. Although further validation by independent replications in humans is required and more work needs to be done to clarify the mechanisms involved, these results suggest an interesting novel avenue for further exploration and provide first promising implications of βB2-crystallin function in the human brain.
